# Continuous Production of Macroporous Films: an Alternative to Breath Figure Assembly

**DOI:** 10.1038/s41598-017-08027-5

**Published:** 2017-08-14

**Authors:** Nazia Noor, Joachim Koll, Clarissa Abetz, Heiko Notzke, Volker Abetz

**Affiliations:** 1 Helmholtz-Zentrum Geesthacht, Institute of Polymer Research, Max-Planck-Str.1, 21502 Geesthacht, Germany; 2 University of Hamburg, Institute of Physical Chemistry, Martin-Luther-King-Platz 6, 20146 Hamburg, Germany

## Abstract

Despite the need for sophisticated instrumentation, breath figure assembly (BFA) methods are restricted to produce macroporous films on a tiny scale so far. The current study narrates the fabrication of macroporous films in hollow fiber geometry which extends to adopt the method for continuous production of isoporous surfaces from commercially available low-priced polymer materials. The fabrication of the films in the hollow fiber geometry is carried out by a co-centric quadruple orifice spinneret through which four different liquids are co-extruded simultaneously: bore fluid (to fill the lumen of the fiber), support layer solution, glycerol, and an isoporous film forming solution through the outer most orifice. The extruded entities plunge into a coagulation bath after passing a definite air gap. The implementation of the concept of diffuse-in, droplet formation, and then condense-out behavior of glycerol in a co-extrusion method of hollow fiber spinning makes macroporous film formation possible in an interminable way sidestepping the use of breath figure assembly method. Moreover, the continuous film formation by the proposed mechanism is also authenticated in flat sheet geometry by employing two casting blades in a casting machine. The structure of the films is analyzed by scanning electron microscopy (SEM).

## Introduction

Porous polymeric films have earned an enormous attention in research because of their significant potential in many fields. This type of films extends their adoption as supporting media in tissue engineering, inorganic growth templates, optical materials, antireflection coating, catalysis, bio or gas sensoring, dielectric materials for electronic devices, stamps for soft lithography, etching masks, etc. Many techniques are available nowadays to create surfaces with ordered uniform pores of nanometer to micrometer sizes. Foremost conceivable techniques are lithography, electron beam sculpting, colloidal templates, emulsion, breath figure assembly (BFA), block copolymer self-assembly, track etching, non-solvent induced phase separation of self-assembled block copolymers, etc^1–8^. Fabrication of many functional nanoporous materials are guided by nanoarchitectonics which spans its field from soft templating to hard templating. Monolayer or multilayer self-assembled lipids or the assembly of polymeric micelles to direct the porous inorganic structures, metal-organic frameworks (MOFs) have been of high research interest as well in recent years for the foreseeable utilization of the produced porous structures in fuel cells, separation technologies, catalysis and many more in near future^9–14^.

Among all the methods for formulating ordered macroporous films, BFA is a captivating root because of its simplicity and cost effectiveness^15–18^. Research on isoporous structure formation from both homopolymers and block copolymers had been clutched on this method for the last two and half decades after this nature inspired phenomenon first got recognized in the work of Widawski *et al*. in 1994^19^. By the BFA method regular arrangements of micrometer sized pores are created by templating the droplets of water formed on cold surfaces. Even though this method does not require any sophisticated equipment, it requires steady state humidity all over the film on which the macroporous structure of high regularity can be built up on. However, the mode of processing does not promise yet a large scale production of films with uniformity in the pore sizes. A continuous process of making honeycomb structures in flat sheet geometry from commercially available polymers was first disclosed by FUJIFILM Corporation which includes the requirement of control over humidity around the production line as well and the final film was obtained after going through several steps^20^. Moreover, the ordered hexagonal structure formation in a dry environment reported before involved the spin coating of the water containing solution. But the spin coating method is limited by the range of area of the porous film it can produce in a single shot^4^.

Along with the flat sheet geometry porous films need to be framed on substrates with cylindrical, spherical, or concave surfaces for many applications, too. Contriving macroporous structure on a non-planar surface is still a challenge even in a small scale and the strategy of transferring porous film on curved surface requires sophisticated arrangement. Some attempts to fabricate porous structures on non-planar substrates by the BFA method involved macro-patterned thin films on a bas-relief pattern^21^, transfer of the colloidal monolayer on a curved surface^22, 23^, casting of a film forming solution on TEM grids, sugar crystal, or patterned silicon wafer^24–26^, combining the breath figure assembly with eletrospinning^27^, and etc. But all these approaches also face the challenge to produce isoporous structures on more than a square centimeter scale in one step.

Recently Wang *et al*. introduced a method for creating an isoporous surface by spin coating of the pore forming material on a porous substrate filled with glycerol and the pores of a micrometer range formed by templating the droplets of glycerol^28^. However, large surfaces of isoporous films cannot be obtained by the spin coating. Widening this pore formation approach to processing methods different from spin coating would allow porous film formation in different geometries in a continuous manner beyond the BFA method.

In this study we report on the formation of macroporous films in hollow fiber geometry from commercially available polymers in a continuous process. For doing so we combined the knowledge of hollow fiber spinning by non-solvent induced phase separation (NIPS) with the pore formation mechanism by diffuse-in and condense-out behavior of glycerol. The means applied to this work are comprised of some essential points: the spinning procedure requires a quadruple orifice spinneret where four different die gaps are designated to four different entities which are co-extruded by the dry-jet wet spinning method, the macroporous film in hollow fiber geometry is fabricated by the concept of diffuse-in condense-out behavior of glycerol, the macroporous film contains a support layer in the spinning stream to give mechanical integrity to the film for its handling afterwards.

The main focus of our study is put on the dry-jet wet spinning method of hollow fiber production. In such a method, one polymer solution (for single layer hollow fiber)^29, 30^ or, several solutions (for multiple layer hollow fiber)^31^ and the bore fluid are extruded synchronously through the different annular gaps of a spinneret. The bore fluid used in such method usually is a precipitant for the spinning solution/s but miscible with the solvent or solvents used in the spinning solution/s. After exiting from the spinneret the bore fluid gets into contact with the spinning solution and starts to solidify the spinning solution from the lumen side of the fiber. The partially solidified fiber enters into the coagulation bath after passing a definite distance in the air. The coagulant used in the coagulation bath is a precipitant for the spinning solution/s and miscible with the solvent/s of the spinning solution/s. After complete precipitation and solidification, the fibers are taken out of the bath and dried in air. The compositions of the bore fluid, the spinning solutions, and the coagulation bath determine the phase separation path of the spinning solution/s which brings about different morphologies in the hollow fiber but this discussion is out of the focus of our present study. Supplementary Fig. S1 depicts the spinning method schematically with a longitudinal and cross sectional view (end of the spinneret) of the quadruple orifice spinneret used in our study. This type of spinneret was employed for fabricating ceramic hollow fibers for solid oxide fuel cells and polymer hollow fibers for membranes^32–34^. Here the quadruple orifice spinneret serves the purpose of co-extruding bore fluid, support layer forming solution, glycerol, and macroporous film forming layer solution (through the orifice 1, 2, 3, and 4, respectively in Supplementary Fig. S1). The dimensions of the die gaps at the spinneret end are listed in Supplementary Table S1.

**Figure 1:**
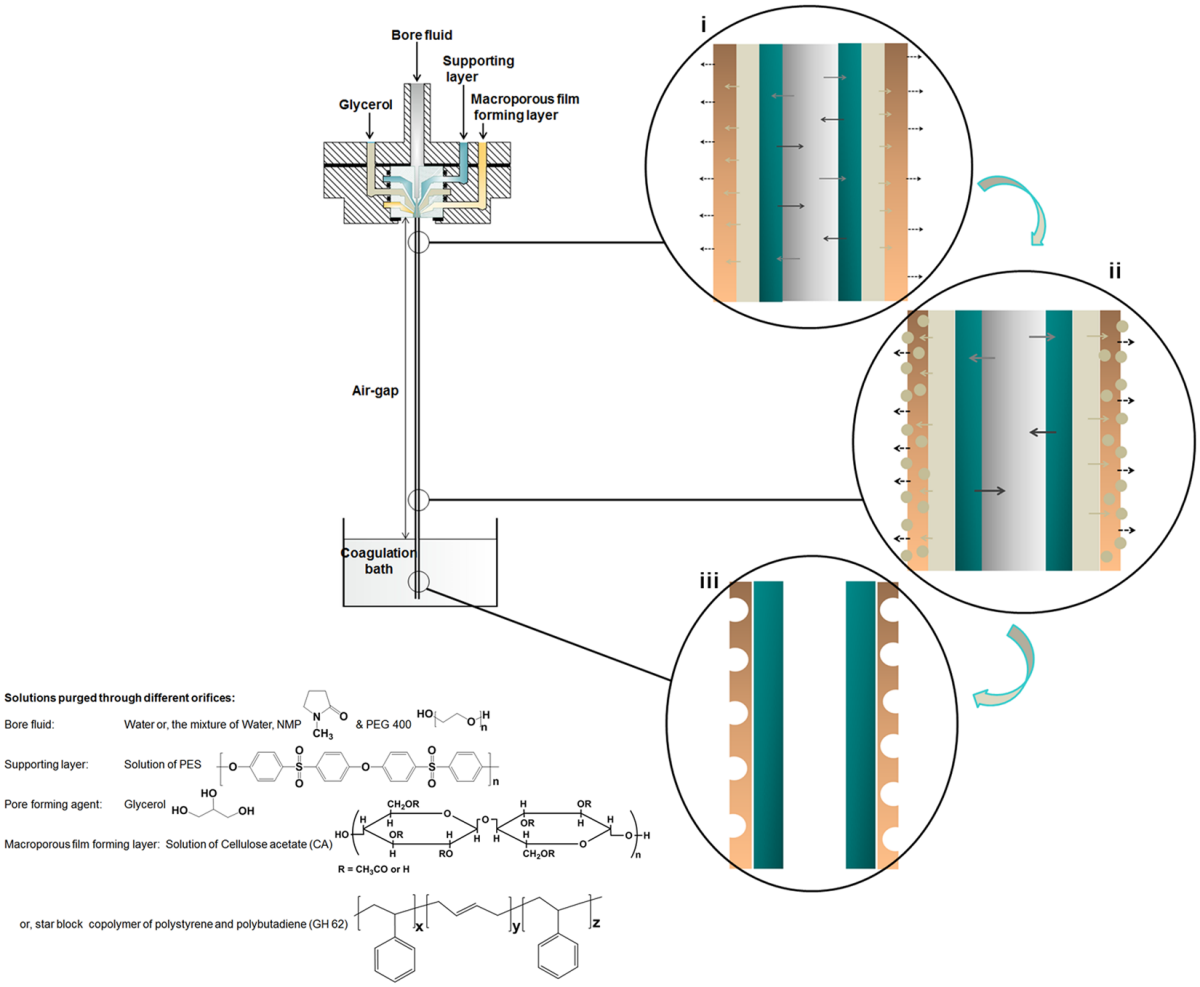
Left part of the picture shows the dry-jet wet spinning method for hollow fiber production mentioning the chemical structures of the materials purged through the different orifices of the spinneret. Right part of the picture depicts the mass transfer phenomena in the hollow fiber; i Solvent and non-solvent exchange between bore fluid and the support layer solution, evaporation of the volatile solvent from the porous film forming layer and meanwhile glycerol diffusion in to the porous film forming layer. ii Solvent and non-solvent exchange between bore fluid and supporting layer solution continues, glycerol gets saturated in the solvent of the film forming solution and forms droplets which condense out afterwards. iii Porous film formed in hollow fiber geometry with support layer underneath. Since different polymers are used for preparing supporting layer solution and the porous film forming layer solution, these two layers typically delaminate from each other

The macroporous structure formation in hollow fiber geometry by templating the glycerol droplets is illustrated in Fig. 1. The porous film forming solution contains a homopolymer or a block copolymer in a highly volatile solvent. After exiting from the spinneret, the volatile solvent starts to evaporate from the surface of the film forming solution and brings down the temperature. Meanwhile glycerol starts to diffuse into the film forming solution. The solvent of the support layer solution exchanges with the non-solvent of the bore fluid in the meantime and the solidification of the support layer solution starts which gives rigidity to the hollow fiber along the spinning axis (Fig. 1i). When glycerol reaches its saturation level in the solvent of the film forming solution, it forms droplets and condenses out of the film. These droplets act as template for the pore formation on the surface of the film and the film forming solution commences to solidify around the glycerol droplets (Fig. 1ii). The hollow fiber immersed into the coagulation bath and gets precipitated after the exchange of solvents of the support layer solution, rest solvent in the film forming solution, and glycerol with water accomplishes (Fig. 1iii). After drying this fiber, a thin polymeric film in hollow fiber geometry with macroporous structure on its surface is obtained and this layer encircles the support layer hollow fiber.

**Figure 2:**
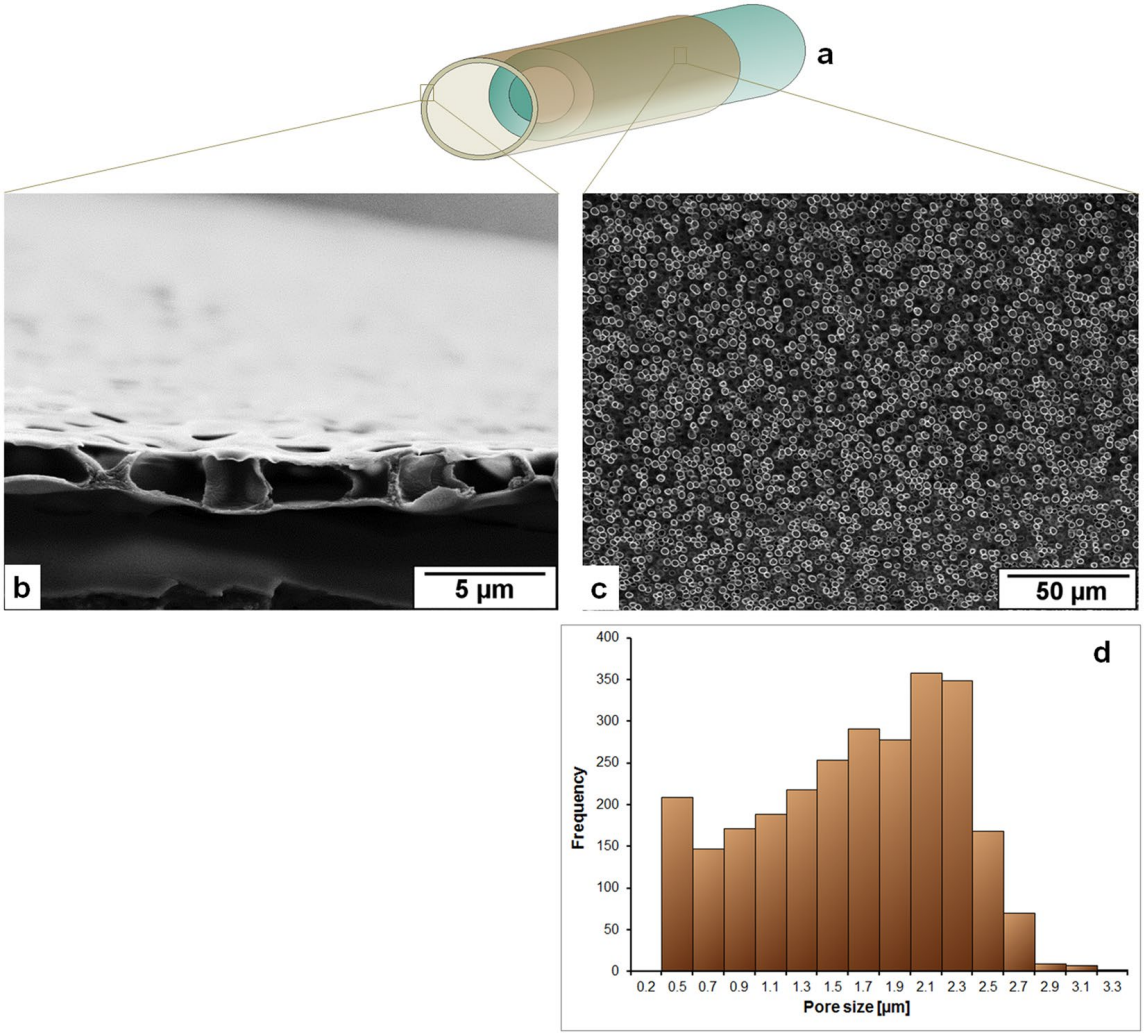
Film forming solution: 8% CA in 1,4-dioxane (Group 1 in Table 1); (**a**) A schematically drawn hollow fiber where the macroporous outer layer film encircles the support layer hollow fiber, (**b**) Cross sectional view of the porous film under scanning electron microscope (SEM), (**c**) Surface of the film under SEM, (**d**) pore size distribution [2724 number of pores in 39.54 × 10^3^ µm^2^ of the measured area] of the surface of the film forming layer measured by IMS V15Q4 (Imagic Bildverarbeitung AG, Glattbrugg, Switzerland).

**Figure 3:**
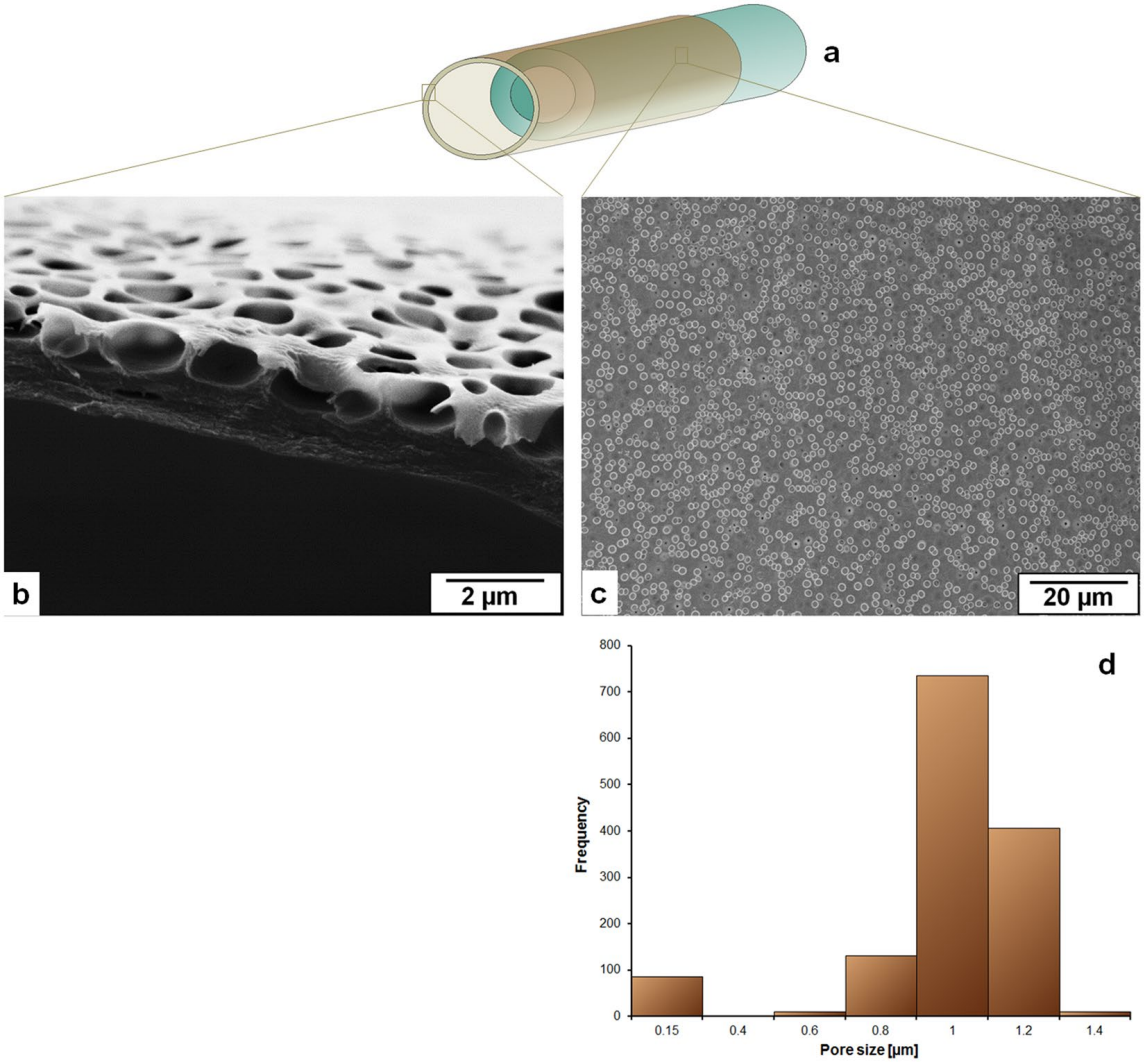
Film forming solution: 12% CA in 1,4-dioxane (Group 2 in Table 1); (**a**) A schematically drawn double layer hollow fiber where the macroporous outer layer encircles the support layer hollow fiber, (**b**) Cross sectional view of the porous film under scanning electron microscope (SEM), (**c**) Surface of the film under SEM, (**d**) pore size distribution [1384 number of pores in 10.04 × 10^3^ µm^2^ of the measured area] of the surface of the film forming layer measured by IMS V15Q4 (Imagic Bildverarbeitung AG, Glattbrugg, Switzerland).

Two different commercial polymers are chosen to validate our process for macroporous film formation. One is cellulose acetate (CA 398–30 Eastman) and another one is a star block copolymer of polystyrene-*b*-polybutadiene-*b*-polystyrene (PS-*b*-PB-*b*-PS) (StyroClear^®^ GH 62 from BASF). Since the support layer solution is employed only as a guide in the spinning line for the macroporous film forming layer, there is a broad variety of the bore fluid and the support layer solution which can be chosen by considering the phase separation behavior of the support layer solution. Depending on the intended application of the macroporous film this layer can be removed later on. In principle also ceramic materials can be used as a support layer, but for the ease of extrusion with our existing spinning facilities we have applied only a polymer solution for this purpose. The experiments are divided into three groups according to the combination of solutions chosen for the spinning. They are listed in Table 1 with the designated orifice of the quadruple orifice spinneret they are purged through and the spinning parameters are summarized in Table 2. All spinning experiments were done in an environment where temperature varied from 20–30 °C and relative humidity was 21–33%. The scanning electron micrographs of the outer surface and the cross section of the films from the three different groups of experiments (Group 1, 2, 3 in Table 1) are presented in Figs 2, 3 and 5, respectively.

**Table 1:** Solutions purged through the different orifices of the spinneret.

Group	1	2	3
Bore fluid (Orifice 1)	Water/NMP/PEG400 (40/30/30)^a^	Water (100)^a^	Water/NMP/PEG400 (40/30/30)^a^
Support layer solution (Orifice 2)	PES/NMP/PEG400/Water (16/40.5/40.5/3)^a^	PES/NMP/PEG400/Water (16/40.5/40.5/3)^a^	PES/NMP/PEG400/Water (16/40.5/40.5/3)^a^
Pore former (Orifice 3)	Glycerol	Glycerol	Glycerol
Film forming solution (Orifice 4)	CA/1,4-dioxane (8/92)^a^	CA/1,4-dioxane (12/88)^a^	GH 62/THF (12/88)^a^

^a^The ratios of the components in the solutions are expressed in wt%.

**Table 2:** Spinning parameters for the experiments of Group 1, 2, and 3 from Table 1.

	Flow rate of Bore fluid (in g min^−1^)	Flow rate of Support layer solution (in g min^−1^)	Flow rate of Glycerol (in mL min^−1^)	Flow rate of Film forming solution (in mL min^−1^)	Air gap (in cm)
Group 1	1	2	0.1	0.1	10
Group 2	1	2	0.1	0.1	
Group 3	1	2	0.05	0.05

Figure 2 shows that 8% solution of cellulose acetate (CA) in 1,4-dioxane as a film forming solution creates pores on the surface with a broad distribution of pore diameters from less than 1 µm to more than 2.5 µm and the surface porosity from the analyzed section is found about 15%. The average film thickness is 2.3 µm. Moreover, the pore depth is almost equal to the film thickness. As the concentration of cellulose acetate increases from 8% to 12% (Fig. 3) the pore sizes accumulate in a narrower distribution mainly with diameters from 0.9 to 1.3 µm. The surface porosity measured in the analyzed section is about 10%. The average film thickness gets to 2 µm and the pore depth is about half of the film thickness. Although the results shown in Figs 2 and 3 are the outcome of same spinning parameters (listed in Table 2), the reason for the different pore sizes and depths may lie in the solution properties and that can be pointed as follows:

1. In spite of using the same solvent higher concentration of polymer decreases the surface tension of the solution which means that the difference in surface energy of glycerol and the film forming solution is higher when 12% of CA solution is used compared to 8% CA solution (surface tension of the solutions, solvents, and glycerol are listed in Supplementary Table S2).
2. If the 8% concentration of CA is too low to stabilize the glycerol droplets then the coalescence of droplets can lead to a broader distribution of pore size.
3. Glycerol dissolves in 1,4-dioxane but aggregates into droplets above a certain concentration and the higher concentration of polymer in 1,4-dioxane may develop higher resistance for the growth of the droplets to the surface.

A decrease of pore size with increasing solution concentration was also observed in the previous work by Wang *et al*. where pores formed on the spin coated polymer solution templating the glycerol droplets^28^. But as reported in several works on pore formation by breath figure assembly^15^, the chances to have the reverse trend in pore size with increasing solution concentration can also not be nullified with this present mechanism. Different combinations of polymers and solvents, molecular weight of the polymer, structure of the polymer, solution concentration can be of further research interest for tuning the size and the order of the pores on the film surface.

Glycerol has limited solubility in 1,4-dioxane^28^ (used for CA solution) and in tetrahydrofuran (THF)^35^ (used for Styro Clear® GH 62 solution). In the present work, from the spinneret exit to the coagulation bath these highly volatile solvents evaporate from the film forming solution while diffused glycerol forms droplets in the film forming solution at lower concentrations of glycerol than in pure 1,4-dioxane or THF. Because of the continuous evaporation of the solvent from the macroporous film forming layer, the temperature drops down at the solution-air interface and the glycerol droplets condense out at the surface. If the glycerol droplets are stabilized at the surface then they act as template for the pore formation. The stabilization of glycerol droplets in the surface is also governed by the spinning parameters and this supposition is supported by comparing the surface structures obtained at different air-gaps.

**Figure 4:**
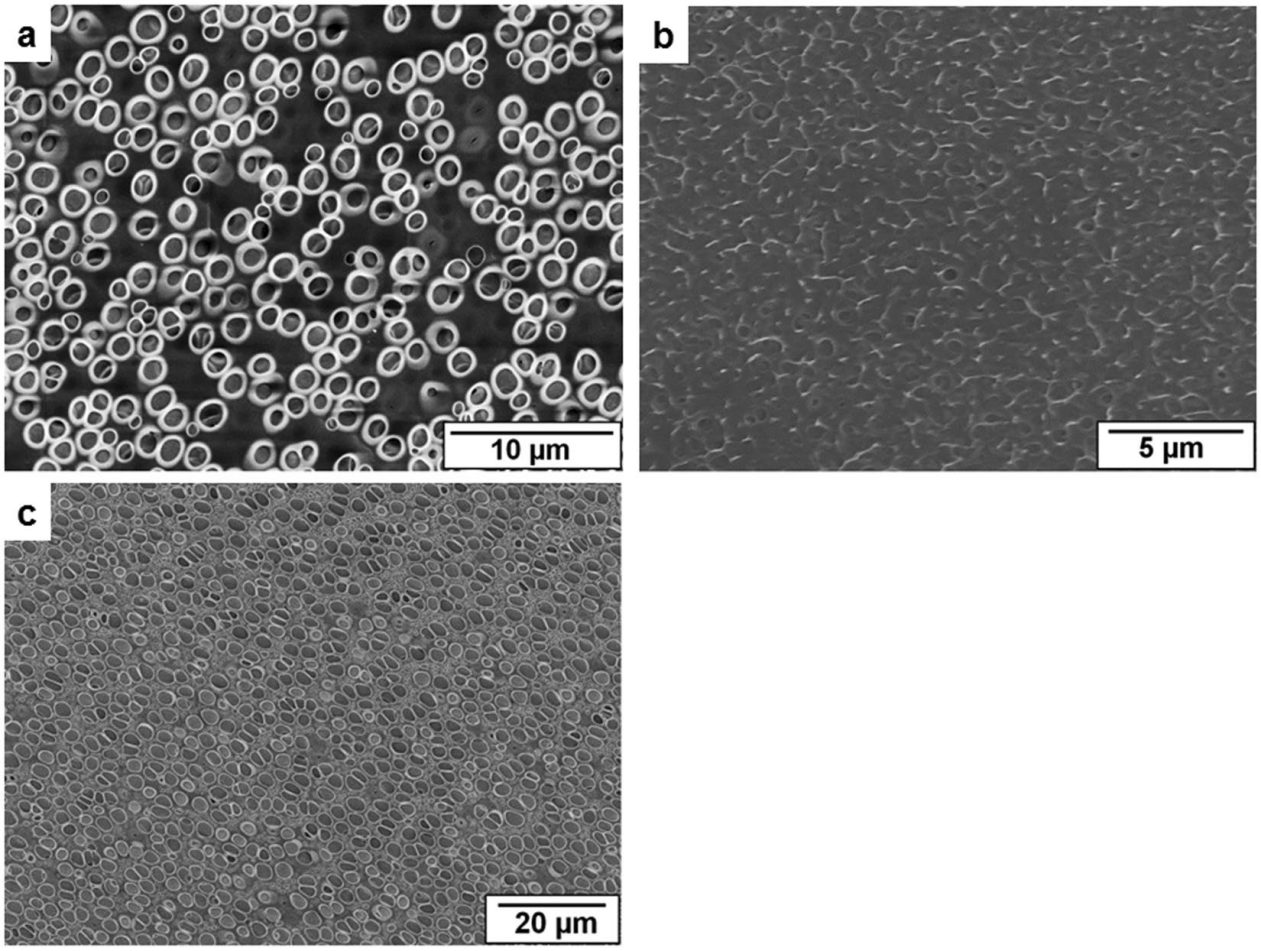
SEM images of the surface of the film spun with air gap of 10 cm (**a**), 25 cm (**b**), and 25 cm with higher flow rates (**c**). Parameters are listed in Table 3.

In Fig. 4 three images of the surfaces are shown which are spun in hollow fiber geometry with different spinning parameters engaging the same solutions for bore fluid, support layer, film, and glycerol. The solution compositions (as of Group 1 in Table 1) and the spinning parameters are listed in Table 3.

**Table 3:** Spinning parameters applied for the experiments of Fig. 4.

Figure no.	Flow rate				Air gap (in cm)
	Bore fluid: (Water/NMP/PEG400 = 40/30/30) (in g min^−1^)	Support layer solution (PES/NMP/PEG400/water = 16/40.5/40.5/3) (in g min^−1^)	Glycerol (in mL min^−1^)	Film forming layer (CA/1,4-dioxane = 8/92) (in mL min^−1^)	
4a	2	2	0.1	0.2	10
4b	2	2	0.1	0.2	25
4c	2	3	0.2	0.2	25

As seen, the surface of the fiber spun with 10 cm air gap distance carries pores (Fig. 4a). With the same flow rates of the bore fluid and dope solutions, increased air-gap (25 cm) i.e. longer time span in the air fades away the pores (Fig. 4b). In contrast, pores are revived on the surface with air gap of 25 cm when the fiber strand plunges into the coagulation bath in a shorter time which is maintained by applying higher flow rate to the extruded solution/s (Fig. 4c). From this observation it is of clear evidence that the pore formation not only is dependent on the solution properties but also on the spinning parameters which results in the optimum time for the pore formation on the surface of the film in hollow fiber geometry.

The pore formation with the residing time of the fiber in the air might be explained as 1,4-dioxane of boiling point 101 °C has slow rate of evaporation and evaporation of 1,4-dioxane promotes condensing out of the glycerol droplets at the cooled surface. But the polymer dissolved in 1,4-dioxane carries some residual solvent at the late stage of solvent evaporation. The residual solvent decreases the glass transition temperature of the solvated polymer film which promotes the relaxation of the chains at the surface. So at this stage if the solvated film stays for long enough time in the air then the glycerol droplets are not stabilized and cavities cannot be formed on the surface templating the droplets. The effect of residual solvent in the polymer film on the pore formation is also evidenced in the BFA mechanism when the evaporation rate of the solvent is slow^36^. Moreover, the effect of increased air gap distance for pore formation is expelled when it is coupled with higher flow rate of polymer solution/s, i.e. having shorter time span of the fiber strand in the air. Furthermore, the surface of the fiber which is spun with longer distance between spinneret and coagulation bath might come up against pore shape stability because of higher elongational stretching of the thin film originated by gravity. That might result in elongated pores (Fig. 4c).

The consequence of using polymers of different structures and behavior is substantiated by choosing a commercially available star block copolymer of polystyrene and polybutadiene (StyroClear^®^ GH 62). Because of its inherent nano-scale heterogeneity this copolymer appears transparent. StyroClear^®^ GH 62 (BASF) is an asymmetric star block copolymer with polybutadiene core and polystyrene external blocks^37^. Such star-shaped block copolymers self-assemble in a variety of ordered structure because of the intramolecular phase separation of the blocks^38, 39^.

**Figure 5:**
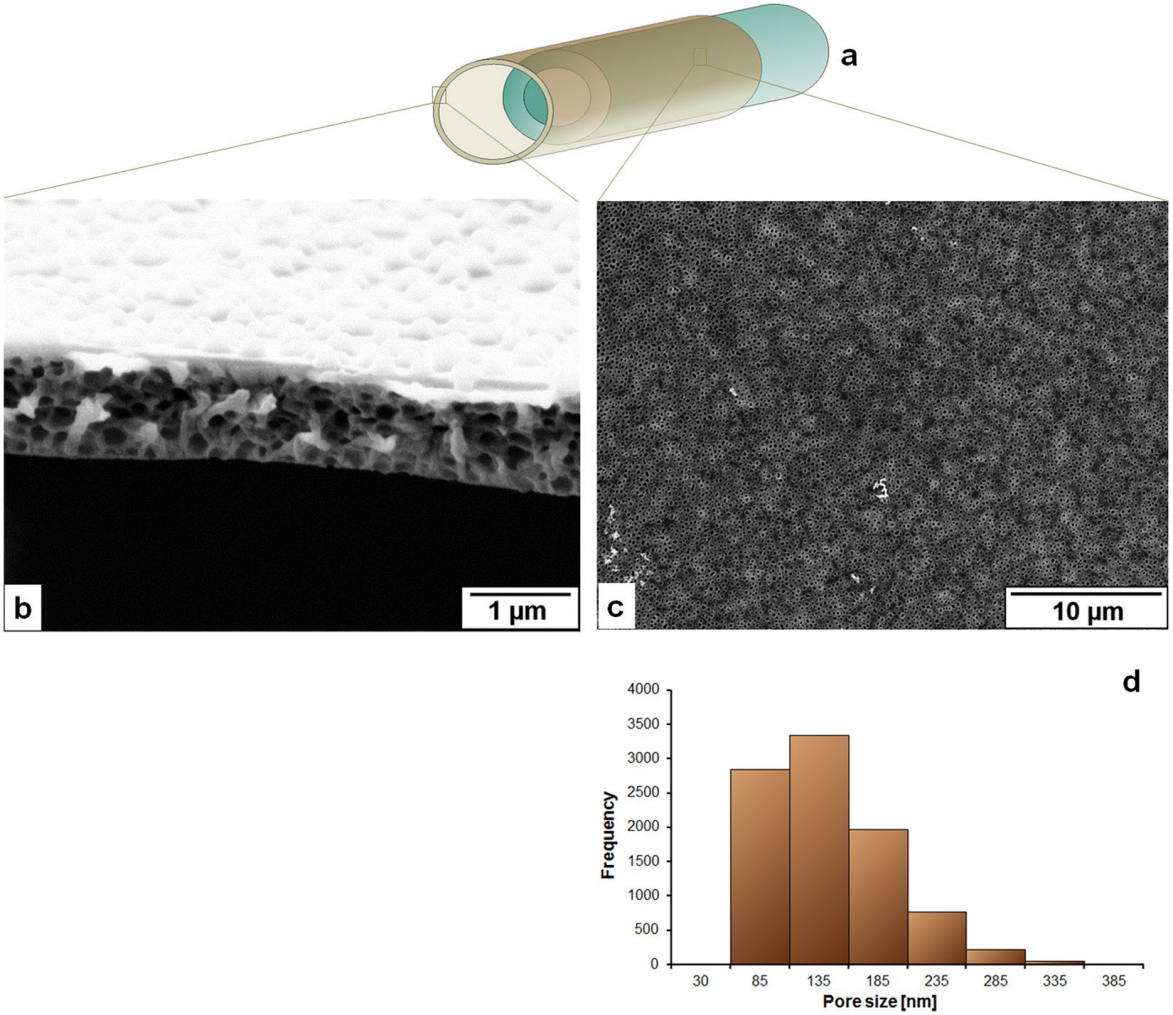
Film forming solution: 12% Styro Clear^®^ GH 62 in THF (Group 3 in Table 1); (**a**) A schematically drawn double layer hollow fiber where the macroporous outer layer encircles the support layer hollow fiber, (**b**) Cross sectional view of the porous film under scanning electron microscope (SEM), (**c**) Surface picture of the porous layer taken by SEM, (**d**) pore size distribution [9206 number of pores in 1.09 × 10^3^ µm^2^ of the measured area] of the surface of the film forming layer measured by IMS V15Q4 (Imagic Bildverarbeitung AG, Glattbrugg, Switzerland).

12% solution of GH 62 in THF by the applied pore forming mechanism coupled with dry-jet wet spinning creates the film with thickness of less than 1 µm and with the surface containing pores of narrower size distribution from 60 nm to 210 nm as majority (Fig. 5). The sharp decrease in pore size using a GH 62 solution compared to a CA solution may be caused by the microphase separation of the block copolymer which favors creation of smaller pore sizes. The previous studies on this commercial star block copolymer showed that it can equilibrate in a lamellar morphology. Obviously the structure formed in our study (Fig. 5) does not resemble the equilibrium morphology. The multilayered pores and the decreased size of pores could be the result of the following reasons: Because of the higher segment density in star polymers they may form a solid polymer layer faster around the glycerol-solution interface. The star block copolymer is dissolved in THF which is a selective solvent for PS blocks and so if the star block copolymer forms quasi-spherical micelles in THF then aggregates of these micelles possess higher rigidity to conserve the glycerol droplets. The larger difference of surface tension between glycerol and THF compared to glycerol and 1,4-dioxane (Supplementary Table S2) leads to a smaller size of the droplets. And if they do not coalesce, then they will form pores by stacking in multiple layers through the film thickness. Similar arguments were advocated also for the porous films which were devised by BFA using both star and nonamphiphilic block copolymers^15, 40, 41^.

**Figure 6:**
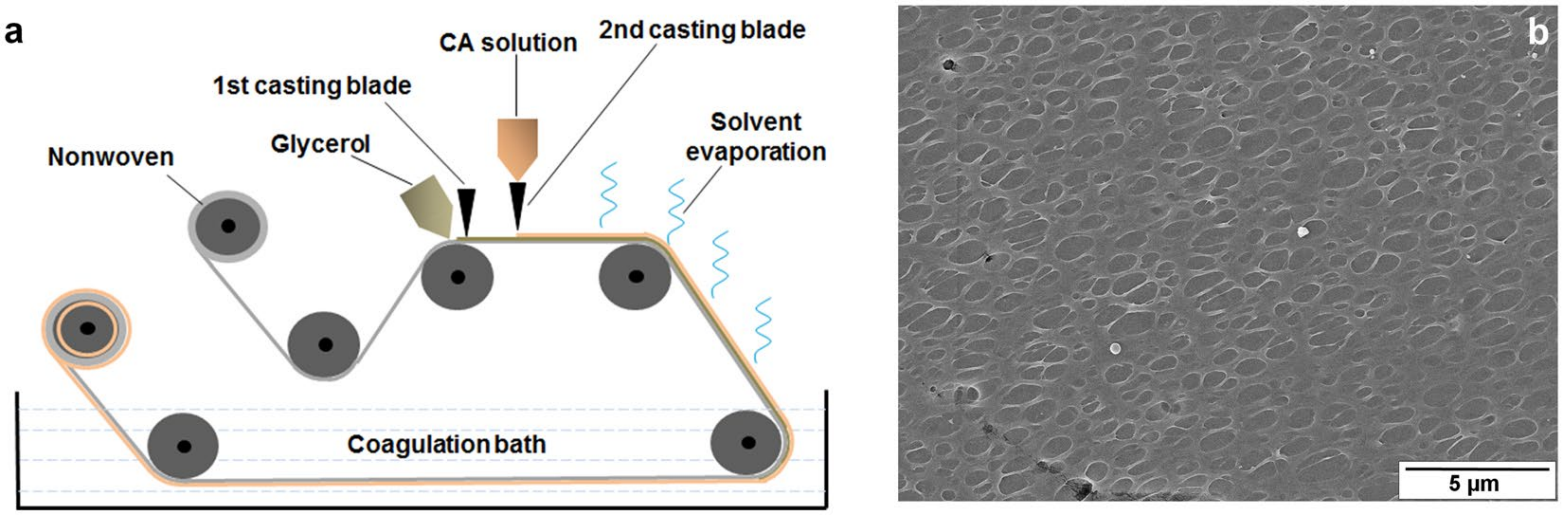
(**a**) Casting machine equipped with two casting blades; first one is for glycerol and the second one is for the film forming solution. (**b**) SEM image of the surface of the film casted with the machine depicted in **(a)** by using 8% CA solution in 1,4-dioxane.

Pore formation in a continuous manner by the mechanism of glycerol diffuse-in condense-out is also possible in flat sheet geometry. An in-house built casting machine (illustrated in Fig. 6a) is used for this purpose. It is equipped with two casting blades. One casting blade is already in-built in the machine and the other one is fixed a few cm ahead of it. The device is equipped with nonwoven support on which the casting is performed. Glycerol is casted on the nonwoven with the first casting blade following the macroporous film forming solution on top of the glycerol layer by the second casting blade. The nonwoven with glycerol and the film forming layer is dipped in the coagulation bath (water) after passing a distance in the air and then collected on a roll. The film forming solution consisted of 8% cellulose acetate (CA) in 1,4-dioxane and the casting was operated at around 20 °C temperature and 28% relative humidity. Although the order in pore size and pore arrangement on the surface is missing, the pore formation by glycerol in a continuous manner is evident in flat sheet geometry, too, as can be seen in Fig. 6b. The optimized combination of the speed of the nonwoven, the gaps between the blades’ ends and the support layer, and solution composition may lead to a better control of the pore arrangement on the surface. Using nonwoven as a support layer causes infiltration of glycerol downward through this support. For avoiding this downward flow of the pore forming component other supports e.g. metallic or polymeric foils can be also used in the proposed instrumentation for porous film formation in flat sheet geometry.

In conclusion, we are introducing a design to explore an alternative way of macroporous film formation other than the conventional ones. In our study we have presented that the pore formation by glycerol droplets welded with the equipment of hollow fiber spinning or flat sheet casting makes possible macroporous film formation in a non-intermittent manner. Analyzing different solutions and spinning parameters (for hollow fiber geometry) or casting parameters (for flat sheet geometry) may lead to uniform pore sizes with their ordered arrangement on the film surface by the described approach in large scale in a single step. The presented method of pore formation can be applied to create ordered assembly of inorganic nanoparticles in nonplanar and planar geometry by using nanoparticle precursors in the film forming solution. Accordingly, the method is expected to offer a continuous process to create micropatterned surfaces which may find their way to applications in catalysis, sensing, templating, cell culture or in microelectronics.

## Electronic supplementary material

Supplementary Information
