# Supplementary material for: Continuous Production of Macroporous Films: an Alternative to Breath Figure Assembly
Source: Sci Rep. 2017 Aug 14;7:8050. doi: 10.1038/s41598-017-08027-5 (PMC5556099; doi:10.1038/s41598-017-08027-5)
Supplement: Supplementary file 1 — Supplementary Information [file 41598_2017_8027_MOESM1_ESM.pdf]

Supplementary Information

Continuous Production of Macroporous Films: an Alternative to Breath Figure Assembly

Nazia Noor[1], Joachim Koll[1], Clarissa Abetz[1], Heiko Notzke[1], Volker Abetz[1,2,*]

[1]Helmholtz-Zentrum Geesthacht, Institute of Polymer Research, Max-Planck-Str.1,

21502 Geesthacht, Germany

[2]University of Hamburg, Institute of Physical Chemistry, Martin-Luther-King-Platz 6,

20146 Hamburg, Germany

*volker.abetz@hzg.de

Contents

S1. Chemicals and Materials

S2. Scanning electron microscopy (SEM)

S3. Dope preparation for hollow fiber spinning and casting

S4. Surface tension measurements

Table

Table S1. Dimensions of the die gaps at the exit of the quadruple orifice spinneret

Table S2. Surface tension of the solutions, solvents, and glycerol

Figures

Figure S1. Dry-jet wet spinning where quadruple orifice spinneret is used for the macroporous film formation in hollow fiber geometry.

S1: Chemicals and Materials

Cellulose acetate (CA 398-30 Eastman) was purchased from Eastman, Styro Clear® GH 62 and (PES) (Ultrason® E6020P) were procured from BASF. Tetrahydrofuran (THF), 1-methyl-2-pyrrolidone (NMP) were purchased from Merck, Germany. 1,4-dioxane (anhydrous 99.8%) Glycerol (anhydrous ≥99%), Polyethylene glycol (PEG400) were purchased from Sigma-Aldrich.

S2. Scanning Electron Microscopy (SEM)

Cross section and the surface of the films were characterized by using scanning electron microscopy (Leo Gemini 1550VP at a voltage of 3-5 kV). For the analysis of the cross section of the films in hollow fiber geometry (which was accompanied by the support layer hollow fiber), hollow fibers were broken in cryogenic condition. All the samples were coated with Pt by using BAL-TEC MED 020 sputtering device and 2 nm coating thickness was controlled by BAL-TEC QSG 070.

S3. Spinning of the hollow fibers

Polymer solutions and the bore fluids were prepared by stirring the respective components with the desired percentages (weight basis) in the sealed glass bottles for overnight. After complete dissolution, solutions were kept in rest for some hours to remove the entrapped air bubbles. After that solutions and the bore fluid were filled in the different designated containers.

For hollow fiber spinning, bore fluid and the solutions for support layer were extruded through the designated orifice of the spinneret by using gear pumps and the flow rates were determined in weight of the solution comes out of the pump in a minute i.e. g/min. Moreover, infusion pumps

were used for purging glycerol and the macroporous film forming solutions through the designated gaps of the spinneret and flow rates were determined in volume of the solutions purged by the pumps in one minute that is mL/min.

For flat sheet casting, CA solution was purged from the container to the designated blade in the casting machine by using gear pumps.

S4. Surface tension measurements

The surface tensions of the solutions, solvents, and glycerol were measured with the force tensiometer K100 from Krüss by Wilhelmy plate method. Measurements were carried out until it fluctuates within ±0.1 mNm^{-1} for the last two readings and each sample was measured for three times.

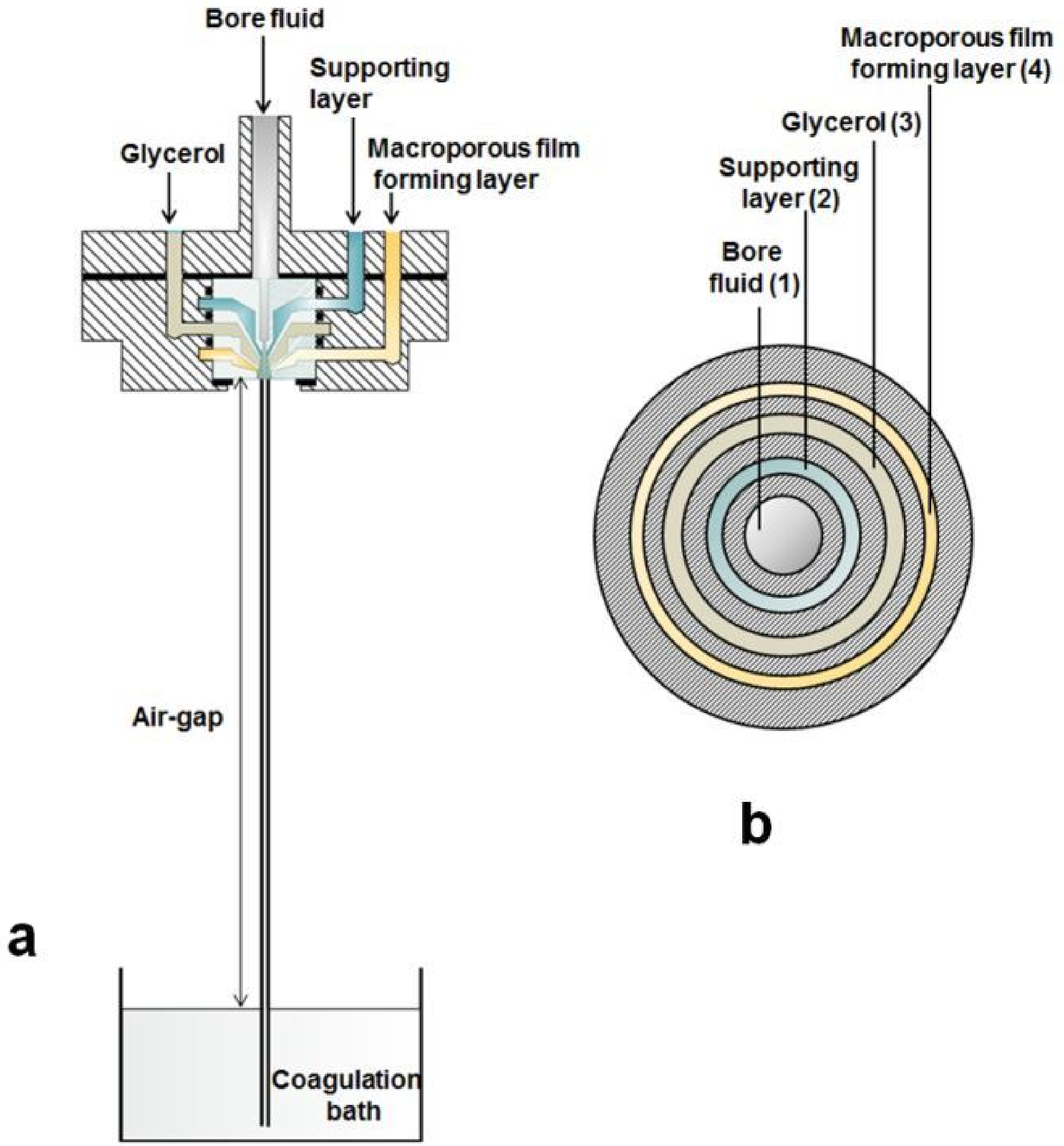

Figure S1. a. Dry-jet wet spinning method of hollow fiber fabrication where a quadruple orifice spinneret is used for co-extruding four different entities through the spinneret. **b.** Cross sectional view of the exit of the quadruple orifice spinneret; 1,2,3,4 are the four orifices of the spinneret which dimensions are listed in **Table S1.**

Orifice no.	Die gap (mm)
1	0.3
2	0.2
3	0.18
4	0.1

Table S1. Dimension of the end of the quadruple orifice spinneret

	Glycerol	1,4-dioxane	THF	8% CA solution in 1,4-dioxane	12% CA solution in 1,4-dioxane	12% GH 62 solution in THF
Surface tension [mN/m]	64.4	33.6	28.8	33.2	29.2	29.1

Table S2. Surface tension of the solutions, solvents, and glycerol
